# Long term results of single high dose Stereotactic Body Radiotherapy in the treatment of primary lung tumors

**DOI:** 10.1038/s41598-019-51900-8

**Published:** 2019-10-29

**Authors:** Luca Nicosia, Chiara Reverberi, Linda Agolli, Luca Marinelli, Vitaliana De Sanctis, Maurizio Valeriani, Mattia F. Osti

**Affiliations:** 1 Department of Radiation Oncology, Sant’Andrea Hospital, “Sapienza” University of Rome, Rome, Italy; 2 Advanced Radiation Oncology Department, IRCCS Sacro Cuore Don Calabria Hospital, Cancer Care Center, via Don Sempreboni 5, 37034 Verona, Negrar Italy; 3 Department of Radiation Oncology, Faculty of Medicine and University Hospital Carl Gustav Carus, Technische Universität Dresden, Dresden, Germany

**Keywords:** Non-small-cell lung cancer, Metastasis

## Abstract

Stereotactic body radiotherapy (SBRT) is a standard treatment for inoperable early-stage NSCLC, with local control rates comparable to surgical series. Promising results have been achieved utilizing a high single-dose schedule. The aim of our study was to evaluate long-term local control and toxicity in a series of patients treated with SBRT delivered in a single dose of 30 Gy. 44 patients affected by early stage NSCLC were treated with SBRT delivered in a single dose of 30 Gy. Survival and prognostic factors were retrospectively evaluated. Median follow-up was 34 months (range 3–81). Three- and 5-year local progression-free survival (LPFS) were 87.8% and 87.8% respectively (median 30 months; range 6–81 months), 3- and 5-year OS and CSS were 64.9% and 36.9%, 80.9% and 65.5%, respectively. Two (4.6%) cases of grade 3 pneumonitis occurred. At the univariate analysis lesion diameter ≤ 25 mm was predictive of better 5-year LPFS (95.8% versus 56.3%; p = 0.003) and 5-year PFS (69.8% versus 27.8%; p = 0.002). The results of our study indicated a high local control, survival and tolerability after a long-term follow-up with the use of SBRT 30 Gy single dose. Further prospective studies could better define the role of this regimen.

## Introduction

Stereotactic body radiotherapy (SBRT) is a recognized and effective therapy in the treatment of inoperable early stage non-small-cell lung cancer (NSCLC), with 3-year local control > 90% comparable to surgical series^1–3^. The high experience accumulated by the radiation oncologists’ community in the treatment of lung tumors and use of modern techniques allow a safe and precise delivery of the treatment, with an incidence of acute severe pneumonitis in the range of 1.6–4.2%^4,5^. Surgery can be considered superior to SBRT because of the surgical nodal sampling, which allows the detection of metastases not recognized by the imaging. Although evidence is conflicting because there has been no direct comparison made through randomized trials, results in terms of local control are nevertheless comparable^4,6–8^.

SBRT is often delivered in multi-fraction regimens (3–10 fractions) however, some studies showed that a single dose can be an attractive regimen, due to the greater convenience for patients, the possibility of reduced positioning error between fractions, the reduction of the slots on the machines and costs, and the possibility to be easily interfaced with systemic therapies^9,10^. Moreover, single high dose radiation may exert their action through tumor vessel damage causing indirect cell death^11^. Despite these advantages the use of single dose SBRT is limited mainly by the fear of severe toxicity and insufficient data on the long-term effectiveness. Reported severe acute toxicity ranged nevertheless from 2.6% to 4.2%^5,12^.

Several publications reported a good tolerability and high rates of local control with the use of a single dose of 30 Gy, but the results after a long term follow-up are scarce and few patients’ or tumors’ characteristics were identified to personalize treatment^5,13,14^. In a previous study we reported the outcome of two single dose SBRT schedules (23 Gy and 30 Gy). The results of the study favored the dose of 30 Gy in terms of local control, with comparable toxicity; therefore this regimen was considered the treatment of choice at our Institution^15^.

We evaluated long-term results of a series of patients affected by early-stage primary NSCLC, treated with SBRT delivered in a single dose of 30 Gy after a long-term follow-up. Local control, survival and toxicity were the primary end-points. Prognostic factors were also assessed.

## Methods

We retrospectively reviewed 44 patients affected by primary lung tumors treated at our Institution between August 2010 and August 2017 with SBRT in a single dose of 30 Gy. According to our internal protocol, patients were discussed by a multidisciplinary team (including thoracic surgeon, oncologist, radiation oncologist, pneumologist, radiologist and pathologist).

Patient selection was based on the following inclusion criteria: (1) performance status ECOG (Eastern Cooperative Oncology Group Criteria) ≤2; (2) no other active sites of disease, loco regional lymph nodes or distant metastasis^3^; ineligible for surgery because of advanced age, comorbidities or refusal of invasive surgery.

When systemic spread was observed during the follow-up period, patients were evaluated to receive: local ablative therapy, systemic therapy or best supportive care by physicians’ evaluation.

Lesions adherent or at least ≤1.5 cm from critical organs at risk or mediastinum were not included in the study to avoid excessive toxicity. Table 1 reported the exact sites of the treated tumors.

**Table 1:** Patients’ characteristics (n = 44).

**Mean age** (years)	75
Range (years)	57–88
**Gender**	
• Male	29 (66)
• Female	15 (34)
**Tumor’s Histology**	
• Adenocarcinoma	17 (38.7)
• Squamous	10 (22.7)
• NSCLC	9 (20.5)
• nd	8 (18.1)
**Clinical stage at diagnosis**	
• cT1a	1 (2.3)
• cT1b	28 (63.6)
• cT1c	11 (25)
• cT2a	3 (6.8)
• cT4	1 (2.3)
Patients’ comorbidities*	
• Cardiological	24 (54.5)
• COPD	20 (45.5)
• Neurological	4 (9)
• Diabetes	4 (9)
• Oncological	2 (4.5)
**Tumor’s location**	
• Right	26 (59)
• Left	18 (41)
• Peripheral	36 (81.8)
• Central	8 (18.2)
**Mean lesion size** (mm/cc)	19.1/4.43
Range lesion size (mm/cc)	9–39/0.51–25.37
**PTV volume**	
• ≤10 cc	21 (47.7)
• 10–16 cc	13 (29.6)
• 16–20 cc	2 (4.5)
• >20 cc	8 (18.2)
**Localization of lesions**	
• SRL	14 (31.8)
• ML	4 (9)
• IRL	8 (18.2)
• SLL	11 (25)
• ILL	7 (16)

*Patients may have more than one comorbidity. COPD: chronic obstructive pulmonary disease, NSCLC: non-small cell lung cancer, SRL: superior right lobe, ML: middle lobe, IRL: inferior right lobe, SLL: superior left lobe, ILL: inferior left lobe.

Pre-treatment evaluation included clinical examination, pulmonary function tests, total body computed tomography (CT) scan, and 18-fluorodeoxyglucose-positron emission tomography (FDG-PET/CT).

The current study was carried out according to the Declaration of Helsinki (1964) and was approved by the Internal Review Board (Department of Radiation Oncology, Sant’Andrea Hospital, “Sapienza” University, Rome). Written informed consent was obtained by all patients.

### Treatment

Details of SBRT planning and delivery at our Institution have been extensively described in previous publications from our Department^5,10^. Briefly, all patients underwent a 4-dimensional (4D) pre-treatment planning CT. The maximum intensity projection was reconstructed using software (Advantage 4D, General Electrics Company, Waukesha, WI) from the 10-phase 4D-CT images and was used to delineate the internal target volume (ITV) from the gross tumor volume (GTV). Planning CT images were matched with diagnostic PET/CT images for the GTV delineation. The planning treatment volume (PTV) was determined by adding 4–5 mm in all directions to the ITV.

The prescribed dose to the PTV was 30 Gy in one single dose (biological equivalent dose 10 [BED10] = 120 Gy) at the 95% isodose with normalization to the maximal dose, for all cases.

Patients’ positioning was verified before treatment using an in-room cone-beam (Kilo-Voltage) CT scan. The treatment was delivered with a Linear Accelerator with 6-MV photons, using 7–9 static non-opposing coplanar fields.

### Follow-up and statistics

Treatment-related adverse effects were assessed at each follow-up according to CTCAE v 4.0. The first follow-up was performed 6 weeks after SBRT with a Chest-CT scan. The following follow-up was performed with a CT scan with contrast medium or FDG/PET-CT every three months for the first two years after SBRT and every six months afterwards.

Local recurrence was defined by the dynamic enlargement of the local tumor on follow-up CTs that continued for at least 6 months and by the increasing of metabolic values at the FDG-PET, routinely used. Moreover, in-field recurrence was defined as any recurrence occurred within the 95% isodose curve and marginal recurrence as any recurrence occurred within the 50% isodose curve.

Survivals were defined as follows: LPFS as the time to occurrence of in-field or marginal regrowth of the disease; PFS as the time to local/distant progression or death; MFS as any site of distant progression (including the ipsilateral lung); CSS was defined as the time to death by cancer or last follow-up; OS as the time to death or last follow-up. Survivals were estimated using the Kaplan–Meier method. Prognostic factors such as age, sex, primary histology, lesion diameter, GTV size, PTV size, type of response and severe toxicity were included in the statistical analysis. Univariate analysis was performed to determine significant prognostic factors using the log-rank test or the Cox method for continuous variables. The multivariate analysis was performed with the multiple logistic regression method and the log-rank test to identify predictive factors; we included in the analysis all the clinically relevant variables. Variables were included in the multivariate analysis according to the correlation at the univariate analysis (P = ≤0.2). The threshold of lesion size related to SBRT response rate and survival was determined with the ROC curve method, calculating the highest product of (sensibility*specificity)^16^. The Statistical analysis was performed using the SPSS statistical software package version 22.0 (SPSS Inc, Chicago, IL). A p-value ≤ 0.05 indicated a significant association.

## Results

### Patients’ characteristics

We treated 44 patients with primary lung tumor. Twenty-nine (66%) patients were male and 15 (34%) were female. Initial stage of disease, patients’ and tumor characteristics are reported in Table 1.

### Local control

**Figure 1:**
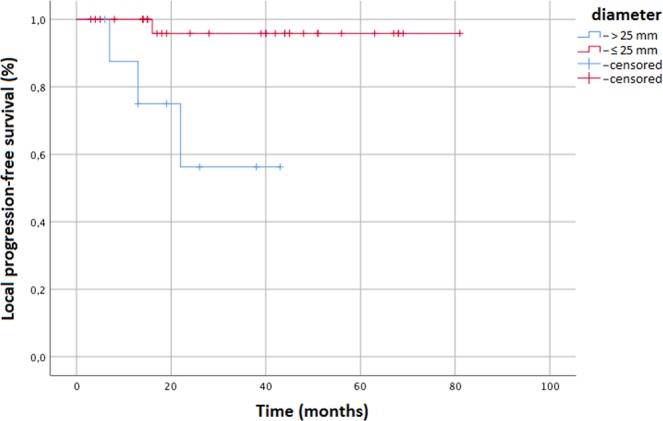
Kaplan Meier curve showing Local progression-free survival stratified to lesion diameter (cut-off 25 mm).

The median follow-up was 34 months (range 3–81). At the time of analysis 4 (9%) patients developed local recurrence as follows: 2 cases had in-field progression (within the 95% isodose curve) and 2 lesions marginal progression (within the 50% isodose curve). The 3- and 5-year local progression-free survival (LPFS) were both 87.8% and 87.8% (median not reached). The 5-year LPFS for lesions ≤ 25 mm diameter was 95.8%, and for lesions >25 mm was 56.3% (p = 0.003) (Fig. 1) (Table 2). At the multivariate analysis lesion diameter ≤25 mm was predictive of higher LPFS (p = 0.005; HR 0.29, i.c. 0.1–0.57). Results of multivariate analysis are reported in Table 3.

**Table 2:** Univariate analysis.

**LPFS (0.003)**	Tumor diameter ≤ 25 mm	Tumor diameter ≤ 25 mm
5 years	95.8%	56.3%
**PFS (0.002)**	Tumor diameter ≤ 25 mm	Tumor diameter ≤ 25 mm
5 years	69.8%	27.8%

**Table 3:** Multivariate analysis.

Factor	*p*	
	LPFS	PFS
Age	n.s.^*^	n.s.
Sex	n.s.	n.s.
Tumor diameter ≤ 25 mm	**0**.**005** (HR 0.29, i.c. 0.1–0.6)	**0**.**004** (HR 0.5, i.c. 0.2–0.9)
Histology adenocarcinoma	n.s.	n.s.

^*^n.s. not significant.

### Pattern of relapse and treatment

Thirteen (32.5%) patients developed local and/or distant progression. Local progression occurred in 4 (9%) patients after a median time of 14 months (range 7–22 months), and was treated as follows: 2 patients received reirradiation using stereotactic technique (50 Gy/5 fractions) and two patients received systemic therapy. One (2.3%) patient developed a relapse to the mediastinal lymphnode and was treated with systemic therapy. Distant progression occurred in 11 (25%) patients after a median time of 22 months (range 2–40 months) and was treated as follows: 6 patients received systemic therapy, 3 patients had oligoprogression other than the previous one site (lung, brain and liver, respectively) and were treated with SBRT plus systemic therapy, and 2 cases received only best supportive care.

### Survival and prognostic factors

At the time of analysis 20 (45.4%) patients had since deceased: 8 (18%) died of systemic progression, 4 (9%) died of chronic obstructive pulmonary disease, 2 (4.5%) of myocardial infarction and 6 (14.5%) of other causes. Dose parameters for patients with myocardial infarction were: Heart V5 3.4% and 5.3%, Hearth V30 0% both; mean lung dose 0.8 Gy and 2.6%; ipsilateral lung V5 6.2% and 7.5%; ipsilateral lung V20 3.1% and 6.2%. The treated tumors were both peripherally located. The first patient had severe hypertension, arterial and mitral valvular sclerosis, and advanced alcoholic liver disease, while the other had severe hypertension.

**Figure 2:**
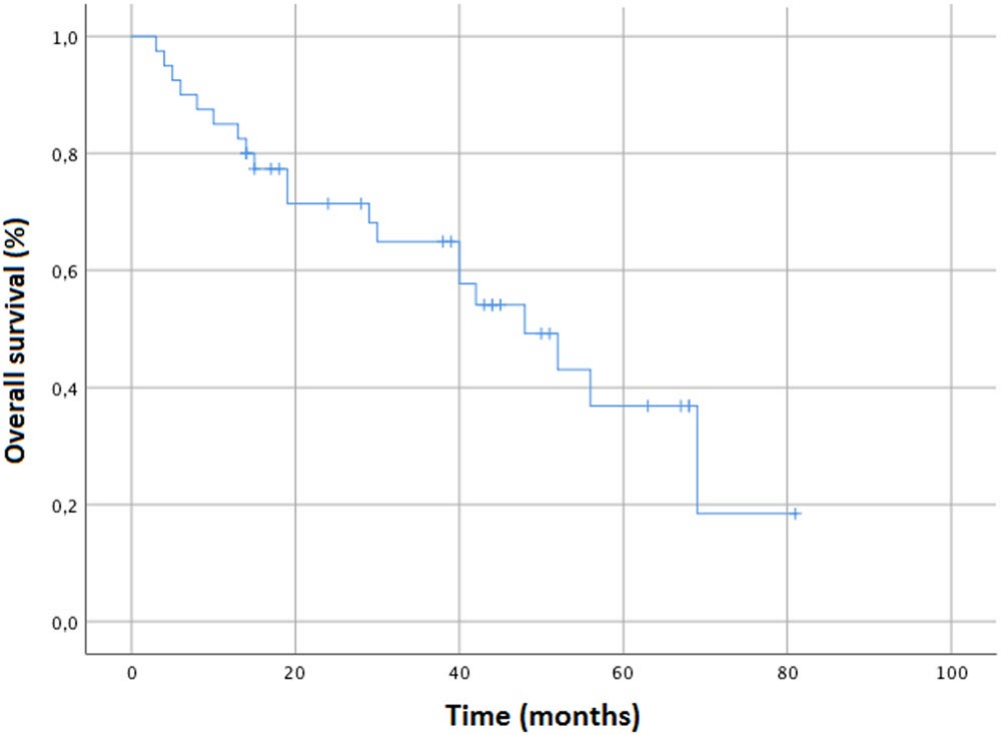
Kaplan Meier curve showing Overall survival for entire patient cohort.

Median overall survival (OS) was 48 months (range 3–81) and 3- and 5-year OS were 64.9% and 36.9%, respectively (Fig. 2). Median cancer-specific survival (CSS) was not reached (range 3–81 months) and the 3- and 5-year CSS were 80.9% and 65.5%, respectively.

**Figure 3:**
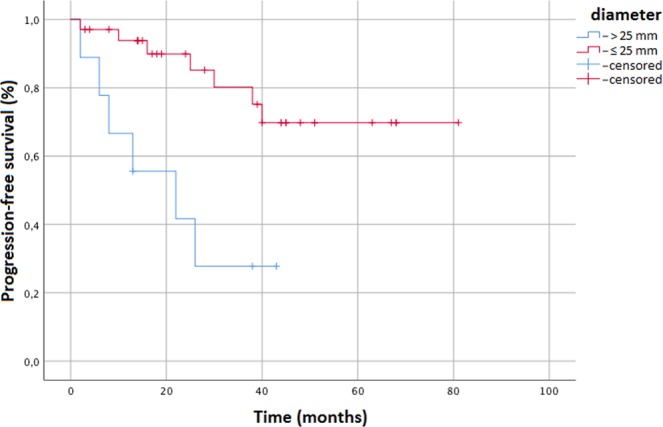
Kaplan Meier curve showing Progression-free survival stratified to lesion diameter (cut-off 25 mm).

Median progression-free survival (PFS) was not reached (range 2–81 months) and the 3- and 5-year PFS were 65.5% and 56.7%. Median metastases-free survival (MFS) was not reached (range 2–81 months) and the 3- and 5-year MFS were 61.4% and 56.7%, respectively. At the univariate analysis lesion diameter ≤25 mm was predictive of higher 5-year PFS (69.8% versus 27.8%; p = 0.002; Fig. 3) and showed a trend towards significance for a higher MFS (69.8% versus 50%; p = 0.053), but did not reflect significant correlations for OS and CSS. At the multivariate analysis lesion diameter ≤ 25 mm was predictive of higher PFS (p = 0.004; HR 0.5, i.c. 0.2–0.9) (Table 3).

### Toxicity

Grade 2 pneumonitis had been detected in 7 (15.9%) patients. Two (4.6%) cases had grade 3 pneumonitis, one of which had a known history of chronic obstructive pulmonary disease and both cases required pharmacological treatment and recovered without sequelae (Table 4); one (2.3%) patient developed grade 2 esophagitis. Moreover Dose-Volume Histograms (DVH) of patients with severe pneumonitis were analyzed and constraints were respected. See dose to critical normal structures in Table 5.

**Table 4:** Acute and late toxicity according to CTCAE v4.0 (n = 44).

Toxicity	Grade 1	Grade 2	Grade 3	Grade 4–5
	N. (%)	N. (%)	N. (%)	N. (%)
**Acute toxicity**				
Pneumonitis	2 (4.6)	7 (15.9)	2 (4.6)	0
Esophagitis	0	1 (2.3)	0	0
Chest wall pain	1 (1.2)	0	0	0
**Late toxicity**				
Lung fibrosis	12 (27.3)	5 (11.4)	1 (2.3)	0

**Table 5:** Dose constraints and dose parameters to critical normal structures.

	Dose constraints	Dosimetric parameters
		mean/range
**MLD**	<15 Gy	1.38 Gy (0.2–3.6 Gy)
**Ipsilateral Lung**		
• V20_Gy_	<10%	1.32% (0.3–3.5%)
• V5_Gy_	<30%	14.5% (3.5–37.5%)
**Controlateral Lung**		
• V20_Gy_	<10%	0%
• V5_Gy_		0.23% (0–5.1%)
**Heart**		
• V30_Gy_	0%	0%
• V5_Gy_	<30%	1.65% (0–27.8%)
**Esophagus**		
• Dmax 5cc	<15.4 Gy	9 Gy (2.3–12.7 Gy)
**Rib**		
• Dmax 1cc	<30 Gy	12 Gy (3–19.2 Gy)
**Spinal cord**		
• Dmax 1cc	<14 Gy	2.96 Gy (0.1–10.1 Gy)

MLD: mean lung dose.

## Discussion

SBRT is a standard treatment for early stage NSCLC, for patients not suitable for surgery or in the case of refusal. SBRT can achieve rates of local control at 5 years of 83.9%, comparable to the 80% of surgical series, as widely demonstrated in many studies^7,8^. On the other hand, SBRT treatment may be characterized by a lower rate of mediastinal nodal control, as compared to surgery, even though the evidences is herein conflicting^17–20^. This might be related to the nodal dissection performed during surgery. Nevertheless, SBRT presents several advantages such as the possibility to be performed also in impaired patients, lower costs and toxicities, low engagement in an outpatient situation, a rapid integration with systemic therapies and parenchymal lung preservation. The optimal schedule is not yet standardized, since many prospective ongoing trials are evaluating it. We previously published a large retrospective series of single dose of 30 Gy SBRT, delivered to lung metastases from different primary tumors, demonstrating a good outcome, especially in small lesions from primary NSCLC^5^. Therefore we analyzed the outcome of a series of early-stage NSCLC treated with stereotactic technique delivered in a single dose of 30 Gy. Long term survival and prognostic factors were evaluated.

Single dose SBRT presents the particular advantage to avoid the intrafraction uncertainties, as compared to multifraction regimens, but 4D simulation and pre-treatment cone beam CT are mandatory, as recommended by the ESTRO ACROP guidelines^1^.

Ma *et al*.^21^ compared 65 small early stage NSCLC treated with 30 Gy via single dose with 94 patients treated with three-fraction schedule SBRT (48–60 Gy), reporting no differences in 2-year local control (87.7% and 86.2% for single-fraction and three-fraction regimen, respectively) and 2-year OS (63.2% and 61.6%, respectively). Only one case of grade 3 pneumonitis occurred in the three-fraction group, with no differences in toxicity rates. Finally the single dose was established as the standard regimen at their Institution.

Videtic *et al*.^13^ treated 82 Stage I medically inoperable NSCLC: 80 patients received single doses of 30 Gy (n = 55) and 34 Gy (n = 25) delivered with SBRT. Unexpectedly the results at 1-year seemed to favor 30 Gy versus 34 Gy in terms of local failure (2% vs. 13.8%), occurrence of distant metastases (10.6% vs. 20.9%), OS (75% vs. 64%) and lung cancer-specific mortality (2.1% vs. 16%). Moreover, no grade 3 toxicity was reported in both arms. Due to the retrospective nature of the study, the authors themselves conclude that these results should be considered with caution.

The phase II trial RTOG 0915 randomized 84 patients affected by stage I peripheral NSCLC to receive SBRT in a single dose of 34 Gy or 48 Gy in 4 fractions. The study fulfilled the primary end-point of safety, with 7.9% of severe acute toxicity in the single dose arm, compared to the 15.8% of multi-fraction schedule. Preliminary results of local control, the secondary end-point of the study, showed 97% and 92.7% at 1 year, respectively, while 1-year OS was 84.6% and 91.1%, respectively. Due to these encouraging results the single dose schedule was considered better tolerated and more effective than the multi-fraction regimen^7^. Nevertheless no factors were evaluated as useful in the selection of patients. Cummings *et al*.^22^ compared 65 patients affected by early stage NSCLC who were treated with SBRT 30 Gy single dose, with 98 patients who were treated with SBRT 50 Gy in 5 fractions. The results of the propensity-matched analysis showed again no differences in 2-year LC (92% versus 82%, p = 0.38; for single dose and five-fractions, respectively, and in the 2-year OS (68% versus 74%, p = 0.18). There was one case of grade 3 pneumonitis in the single dose group, but no differences were observed when compared with the five-fraction regimen.

The long-term results of the current study and the related LPFS at 5 year of 87.8% are in line with previous publications. We observed that most of recurrences occurred during the first 2 years of follow-up that may justify an intensive and multimodal approach.

One of the major concerns regarding the use of SBRT is the possibility of a higher mediastinal nodal relapse, as compared to surgery^4,6^. The cumulative incidence of regional recurrence after SBRT for early stage NSCLC, diagnosed with PET-FDG ranged from 0 to 28.6% with a median incidence of 9.6%^23^. We reported only one case of mediastinal nodal relapse, which can be related to the systematic use of PET in the staging that allowed for a more accurate selection of patients, and the enhancement of detection rates of mediastinal relapse after SBRT^24^.

Local progression in our series occurred in 4 (9%) tumors after a median time of 14 months: 3 out of 4 of these occurred in lesions >25 mm, which can represent a threshold of effectiveness and can be used to select early-stage NSCLCs’ best population suitable for the treatment with 30 Gy SBRT. It is known that smaller lesions respond better to SBRT^1,25–28^, but no studies, to data, have assessed dimensional prognostic factors of response after 30 Gy SBRT in this setting. Moreover some controversies exist regarding the definition of local failure, since some authors define it as any relapse within the same lobe and/or of the treated lesions^7,29^, while others consider it as the sole failure of the treated lesion^30,31^ and this can complicate the comparison and interpretation of the results.

The cut-off of 25 mm represents a novel finding, since patients’ selection is crucial for maximizing treatment outcome. Larger tumors with hypoxic areas may benefit more from a multi-fractions regimen that can directly induce cell death and take partial advantage of the reoxygenation, while smaller tumors may benefit the most from a high dose treatment in a single dose which affects the tumor vessels and indirectly determines cell death^11,31^.

Toxicity of our series was mild and in line with previous publications on 30 Gy single dose treatments, with pneumonitis occurring in 1.7–3.6% of cases^5,13,14,32–35^. We reported two cardiac deaths in patients with severe cardiac and pulmonary diseases. By the DVH analysis and considering the location of the treated lesions away from central organs we did not ascribe these events as treatment-related death, also considering the recent literature on the correlation between dose to cardiac structures and cardiac event^36^.

Some limitations of the present study are the retrospective nature and the small population, while points of strength are the long-term results, the use of multimodal imaging for the staging and during the follow-up, the homogeneity of patients’ characteristics and the identification of a threshold of higher efficacy.

Our study represents the first report on the long-term outcome of a high dose SBRT delivered in a single dose of 30 Gy in primary lung tumors. Smaller tumors can achieve 5- year local control of 95.8% at the cost of very limited toxicity. Prospective studies are warranted to confirm these results and to evaluate possible molecular factors predictive of response.

## Conclusion

Lung SBRT with a single high dose of 30 Gy is safe and effective. Long-term results confirmed its effectiveness with high rates of local control, especially for small lesions. A prospective trial is recommended to better define the therapeutical range of this schedule, including also biological parameters.
